# LncRNA HOXA11-AS Promotes Proliferation and Cisplatin Resistance of Oral Squamous Cell Carcinoma by Suppression of miR-214-3p Expression

**DOI:** 10.1155/2019/8645153

**Published:** 2019-05-28

**Authors:** Xiaoyan Wang, Hong Li, Jing Shi

**Affiliations:** Department of Oral Medicine, The People's Hospital of Shanxi Province, Taiyuan 030012, Shanxi Province, China

## Abstract

Drug resistance to platinum limited therapeutic options for oral squamous cell carcinoma (OSCC). In the current study, we investigated the role of lncRNA HOMEOBOX A11 (HOXA11) antisense RNA (HOXA11-AS) in OSCC resistance to cisplatin (CDDP). We used clinical tissues and OSCC cell lines and induced CDDP resistance in OSCC cells. Gain and loss of function were performed in OSCC-resistant cells. Xenograft mice were also established. HOXA11-AS expression was increased in OSCC clinical tissues and cell lines and upregulated in CDDP-resistant cells. Upregulation of HOXA11-AS promoted proliferation in CDDP-sensitive cells and inhibited CDDP-induced cytotoxicity. In contrast, downregulation of HOXA11-AS decreased proliferation in CDDP-resistant cells and increased CDDP-induced cytotoxicity. Knockdown of HOXA11-AS inhibited the tumor growth in xenograft mice injected by CDDP. Downregulation of HOXA11-AS increased apoptosis and caspase 3 activities in CDDP-resistant OSCC cells. Bioinformatics, reporter assay, and loss and gain of function assay indicated that HOXA11-AS and miR-214-3p could negatively regulate each other. miR-214-3p was decreased in OSCC clinical tissues and cell lines. We further revealed that proto-oncogene serine/threonine-protein kinase (PIM1) was the target of miR-214-3p. PIM1 expression could be negatively regulated by miR-214-3p and positively regulated by HOXA11-AS. Inhibition of PIM1 suppressed anti-miR-214-3p-induced increase of cell proliferation and decrease of apoptosis. In summary, HOXA11-AS was identified to facilitate CDDP-resistance in OSCC and miR-214-3p/PIM1 was found to be the downstream target of HOXA11-AS. The findings highlight the importance of HOXA11-AS/miR-214-3p/PIM1 axis in the drug resistance of OSCC and provide potential targets for improving chemotherapy of OSCC.

## 1. Introduction

Oral squamous cell carcinoma (OSCC) is one of the most common head and neck squamous cell carcinomas (HNSCCs) with a changing molecular and demographic profile [1–4]. OSCC occupies approximately 3% of all recently diagnosed clinical cancer cases [1]. Despite the substantial improvements in multimodality approaches, the cure rate in those patients receiving such treatments is only 50% [5]. To date, the overall 5-year survival rate of OSCC patients is less than 50% [6]. Chemotherapy (such as platinum) is an efficient adjuvant treatment for OSCC patients in some cases. However, drug resistance to platinum limited therapeutic options with a median overall survival time of 6-9 months in OSCC [7]. Thereof, the better understanding of the molecular mechanisms underlying platinum-based drug resistance in OSCC is urgently needed for the efficient therapy of OSCC patients.

Long noncoding RNA (lncRNAs) is a type of nonprotein-coding and functional RNAs that contains more than 200 nucleotides [8, 9]. LncRNA could function to regulate target gene expression through multiple mechanisms, including epigenetic, transcriptional, and posttranscriptional modulation. In recent years, it is shown that lncRNAs plays a role in almost all physiological and pathological processes of the body. More and more evidence has demonstrated that dysregulation of lncRNAs is involved in development of a variety of diseases. In particular, a battery of lncRNAs is dysregulated in the initiation and development of cancer. LncRNAs has been reported to play both an oncogenic and tumor suppressive role [10, 11]. LncRNAs participates in the regulation of multiple cancer-related cellular and molecular processes, including cell cycle transition, cell proliferation, differentiation, apoptosis, invasion, and migration [12–14].

LncRNA HOMEOBOX A11 (HOXA11) antisense RNA (HOXA11-AS) has been verified to participate in cancer development. Based on previous reports, we concluded that HOXA11-AS function as an oncogenic or tumor promotive regulator in several types of human cancers [15–21], such as colorectal cancer, lung cancer, hepatocellular carcinoma, glioma, breast cancer, renal cancer, ovarian cancer, melanoma, and gastric cancer. HOXA11-AS plays an oncogenic role in the cellular processes of laryngeal squamous cell cancer (LSCC) and serves as a novel marker and a potential therapeutic target in LSCC patients [22]. HOXA11-AS drives cisplatin (CDDP) resistance of human lung cancer [23]. Based on these literatures, we proposed that HOXA11-AS played a role in chemoresistance in OSCC.

In the current study, we designed experiments to investigate the role of HOXA11-AS in OSCC resistance to CDDP, a platinum-based anticancer drug. We showed that HOXA11-AS expression was increased under CDDP-resistant condition in OSCC cells. HOXA11-AS facilitated CDDP-resistance through regulation of miR-214-3p/PIM1.

## 2. Materials and Methods

### 2.1. Clinical Tissue Specimens

This study was approved by the Research Ethics Committee of The People's Hospital of Shanxi Province. 31 patients with OSCC, who received surgical treatment in The People's Hospital of Shanxi Province from April 2015 to March 2016, were recruited in this study. OSCC tumor tissues and normal adjacent squamous epithelium were collected. All participants signed informed consent prior to using the tissues for scientific research. The tumor tissues were immediately frozen in liquid nitrogen and then stored at -80°C for further analysis.

### 2.2. Cell Culture

The normal human oral keratinocyte (NHOK) and OSCC cell lines, including TSCCA, CAL-27, SCC-9, and Tca8113, were ordered from the Cell Bank of Type Culture Collection of Chinese Academy of Sciences (Shanghai, China). TSCCA and CAL-27 cells were exposed to gradually increasing doses of CDDP to establish CDDP-resistant OSCC cells (TSCCA-CDDP and CAL-27-CDDP). All cells were maintained in DMEM medium supplemented with 10% FBS (Invitrogen, Carlsbad, CA, USA), 100 U/mL penicillin, and 100 mg/mL streptomycin in a humidified atmosphere of 5% CO_2_.

### 2.3. Plasmid Transfection

SiRNAs against HOXA11-AS (si-HOXA11-AS; 5′-CTACCATCCCT GAGCCTTA-3′) and negative control siRNA (si-NC), sh-HOXA11-AS (CACCAGGCCAAGTCCGAGTTCCATTTCTTCGAAAAGAAATGGAACTCGGACTTGGCC) and sh-NC were all purchased from GenePharma. PIM1 shRNA lentiviral transduction particles were performed from Sigma-Aldrich (SHCLNV-NM_002648). The sequence of HOXA11-AS cDNA was amplified and cloned to pcDNA3.1 (Invitrogen, Carlsbad, CA, USA). pre-miR-214-3p, anti-miR-214-3p, or control miR (miR-NC and anti-miR-NC) were obtained from Invitrogen Technology (Ambion, Austin, TX, USA). Transfection was performed using lipofectamine 2000 (Invitrogen, Carlsbad, CA, USA) according to the manufacturer's instructions. Cells were collected and transfection efficiency was examined 48h after transfection.

### 2.4. Cell Proliferation

Cell proliferation was analyzed by using cell counting assay Kit-8 (CCK-8) (EnoGene, China) as per the manufacture's protocols. After the transfection and incubation, cells were incubated with serum-free culture medium containing 10% CCK-8 solution at 37°C for 1-2 h. The absorbance of each well at 450 nM was measured at 0, 24, 48, and 72h after transfection using a microplate reader (Bio-Rad Laboratories, Inc., CA, USA).

### 2.5. Determination of IC50 of CDDP

CDDP-induced toxicity in OSCC cells was evaluated by measurement of the IC50 value using CCK-8 assay. Briefly, TSCCA-CDDP and CAL-27-CDDP cells were seeded in 96-well plates, treated with indicated concentrations of CDDP and then the cell viability was measured using the CCK-8 kit according to the manufacturer's protocols.

### 2.6. Luciferase Reporter Assay

The wild-type HOXA11-AS (WT), mutant HOXA11-AS (MUT), PIM1 (WT), and PIM1 (MUT) containing the binding site of miR-214-3p were incorporated into the pmirGLO dual-luciferase vector (Promega, Madison, WI, USA). HEK293T cells were cotransfected with pmirGLO-HOXA11-AS-WT or pmirGLO-HOXA11-AS-MUT, pmirGLO-PIM1-WT, pmirGLO-PIM1-MUT, and miR-214-3p mimics or negative control by Lipofectamine 2000. 48 h after transfection, the luciferase activities were measured with Dual-Luciferase Reporter Assay System according to the manufacturer's instructions. Relative luciferase activity was expressed as normalization of renilla luciferase activity to firefly luciferase activities. Triplicate independent experiments were performed to measure luciferase activity.

### 2.7. Flow Cytometric Analysis of Apoptosis

Apoptosis was evaluated using flow cytometric analyses with TUNEL Apoptosis Detection Kits (Roche, Switzerland) according to the manufacturer's instructions. Triplicate independent analysis was performed.

### 2.8. Xenograft Mice Model

All animal experiments were approved by the Institutional Animal Care and Use Committee of The People's Hospital of Shanxi Province and in accordance with ARRIVE and NIH guidelines for animal welfare. 12 male BALB/c nude mice were obtained from Animal Center of Shanxi Medical University and maintained under pathogen-free conditions. TSCCA-CDDP cells (1 × 10^7^) infected with sh-HOMA11-AS or sh-NC were suspended in 100 *μ*L medium and then subcutaneously injected into the right flanks of mice. 7 days after the injection, the mice were injected with 4 mg/kg CDDP or an equal volume of PBS once every 4 days. Tumor growth was measured at indicated time points at 7, 11, 15, 19, 23, 27, and 31 days with a digital caliper. Tumor volumes were calculated as 0.5 × length × width^2^. At the end of the experiment, mice were euthanatized for tumor weight analysis.

### 2.9. RNA Extraction and Quantitative Real-Time Polymerase Chain Reaction

Total RNA of cultured cells was isolated by TRIzol reagent (Life Technologies, Carlsbad, CA, USA) according to the manufacturer's instructions. RNA quantification was performed using a NanoDrop ND-2000 and cDNA was reversely transcribed using the RT reagent Kit with gDNA Eraser (Takara, Dalian, China) according to the manufacturer's protocols. Quantitative RT-PCR was performed using SYBR Green Master Mix (Takara, Dalian, China) on a Bio-Rad system. The expression level of miRNA was examined using Taqman microRNA Reverse Transcription Kit and Taqman Universal Master Mix II with the TaqMan MicroRNA Assay of miRNAs (Applied Biosystems, USA). GAPDH and U6 were used as the internal normalizer. The PCR reaction conditions for all of the assays were 94°C for 30 seconds, followed by 40 cycles of amplification (94°C for 5 seconds, 60°C for 30 seconds, and 72°C for 30 seconds). Relative mRNA level was calculated using the 2^−ΔΔCt^ method. The sequences of primers were as follows: HOXA11-AS forward: 5'-CGGCTAACAAGGAGATTTGG-3' and reverse: 5'-AG- GCTCAGGGATGGTAGTCC-3'. miR-214-3p forward: 5'-ACAGCAGGCACAGACAGG-3' and reverse: 5'- GTGCAGGGTCCGAGGT-3'. GAPDH forward: 5'-GGGAGCCAAAAGGGTCAT-3' reverse: 5'- GAGTCCFTTCCACGATACCAA-3'.

### 2.10. Caspase 3 Activity

Caspase 3 activity was determined using a Caspase 3 Activity Assay Kit (Beyotime Company, China) according to the manufacture's manual.

### 2.11. Bioinformatics Analysis

Star Base v2.0 was used to predict the binding of HOMA11-AS and miR-214-3p. TargetScanHuman 7.1 was used to predict the downstream targets of miR-214-3p.

### 2.12. Statistical Analysis

Data were displayed as means ± standard deviation (SD). Statistical analysis was performed using Graphpad Prism software 6.0. Statistical significance was evaluated using one-way analysis of variance followed by Student-Newman-Keuls test. p < 0.05 was considered to be statistically significant.

## 3. Results

### 3.1. HOMA11-AS Expression Was Upregulated in OSCC Clinical Tissue Samples and Cell Lines

**Figure 1:**
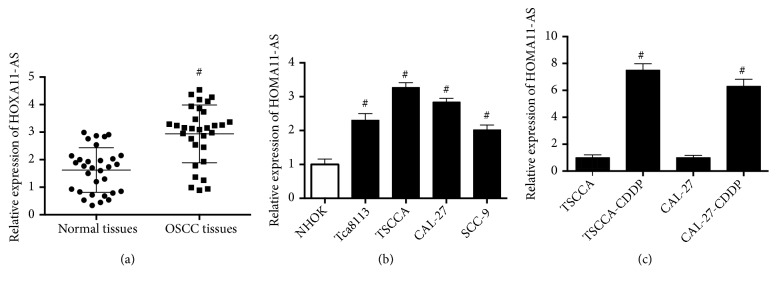
*HOMA11-AS expression was upregulated in OSCC clinical tissue samples and cell lines*. (a) mRNA expression level of HOMA11-AS in tumor tissues and corresponding noncancerous tissues of 31 OSCC patients (p=0.016). (b) mRNA expression level of HOMA11-AS in normal human oral keratinocyte cell line (NHOK) and OSCC cell lines (TSCCA, CAL-27, SCC-9, Tca8113) (p=0.009; p=0.002; p=0.005; p=0.011). (c) mRNA expression level of HOMA11-AS in OSCC cell lines (TSCCA and CAL-27) and their corresponding CDDP-resistant cell lines (TSCCA-CDDP and CAL-27-CDDP) (p=0.0009; p=0.001). #P < 0.05.

The expression pattern of HOMA11-AS in OSCC tissues and cells was examined and the results showed that HOMA11-AS expression in OSCC clinical tissues was significantly higher than that in adjacent normal tissues (Figure 1(a)). Additionally, HOMA11-AS expression in OSCC cell lines, such as TSCCA, CAL-27, SCC-9, and Tca8113 cells, was higher than that in normal human oral keratinocyte cell line (NHOK) cell lines (Figure 1(b)). Moreover, we compared the expression of HOMA11-AS between the CDDP-sensitive and resistant OSCC cells.  Figure 1(c) showed that compared with CDDP-sensitive state, HOMA11-AS expression was increased when the cells were CDDP-resistant. The results suggested that HOMA11-AS upregulation was involved in CDDP-resistance in OSCC cells.

### 3.2. HOMA11-AS Facilitated Proliferation and CDDP Resistance in OSCC Cells

**Figure 2:**
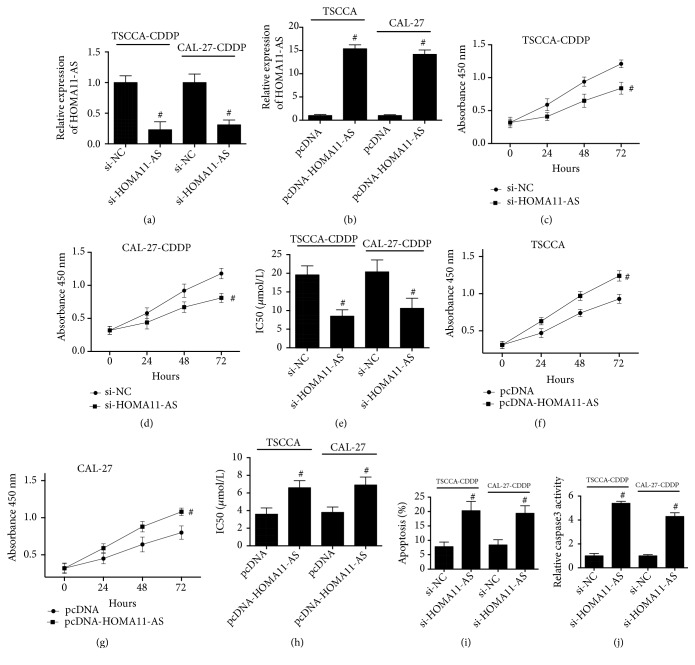
*HOMA11-AS facilitated proliferation and CDDP resistance in OSCC cells*. (a) TSCCA-CDDP and CAL-27-CDDP cells were transfected with si-HOMA11-AS or its negative control, and the transfection efficiency was confirmed by real-time PCR (p=0.021; p=0.024). (b) TSCCA and CAL-27 cells were transfected with pcDNA-HOMA11-AS or its negative control, and the transfection efficiency was confirmed by real-time PCR (p=0.0008; p=0.0009). ((c) and (d)) Cell proliferation in TSCCA-CDDP and CAL-27-CDDP cells transfected by si-HOMA11-AS or its negative control (p=0.017; p=0.022). (e) IC50 value of CDDP in TSCCA-CDDP and CAL-27-CDDP cells transfected by si-HOMA11-AS or its negative control (p=0.025; p=0.031). ((f) and (g)) Cell proliferation in TSCCA and CAL-27 cells transfected by pcDNA-HOMA11-AS or its negative control (p=0.019; p=0.017). (h) IC50 value of CDDP in TSCCA and CAL-27 cells transfected by pcDNA-HOMA11-AS or its negative control (p=0.032; p=0.038). (i) Apoptosis in TSCCA-CDDP and CAL-27-CDDP cells transfected by si-HOMA11-AS or its negative control was determined using TUNEL assay (p=0.029; p=0.028). (j) Caspase 3 activities in TSCCA-CDDP and CAL-27-CDDP cells transfected by si-HOMA11-AS or its negative control were examined using a specific assay kit (p=0.001; p=0.002). #P < 0.05.

To test the hypothesis that HOMA11-AS was a key regulator of CDDP resistance in OSCC cells, we downregulated in CDDP-resistant OSCC cells or upregulated HOMA11-AS expression in CDDP-sensitive OSCC cells. We found that downregulation of HOMA11-AS (Figure 2(a)) inhibited the proliferation of CDDP-resistant TSCCA and CAL-27 cells (Figures 2(c) and 2(d)) and decreased the IC50 value of CDDP toxicity (Figure 2(e)). Upregulation of HOMA11-AS (Figure 2(b)) facilitate the proliferation of CDDP-sensitive TSCCA and CAL-27 cells (Figures 2(f) and 2(g)) and increased the IC50 value of CDDP toxicity (Figure 2(h)). In CDDP-resistant TSCCA and CAL-27 cells, downregulation of HOMA11-AS resulted in a significant increase of apoptosis (Figure 2(i)) and caspase 3 activities (Figure 2(j)). The results demonstrated that HOXA11-AS facilitated resistance to CDDP in TSCCA and CAL-27 cells.

### 3.3. HOMA11-AS Interacted with and Negatively Regulated miR-214-3p in OSCC Cells

**Figure 3:**
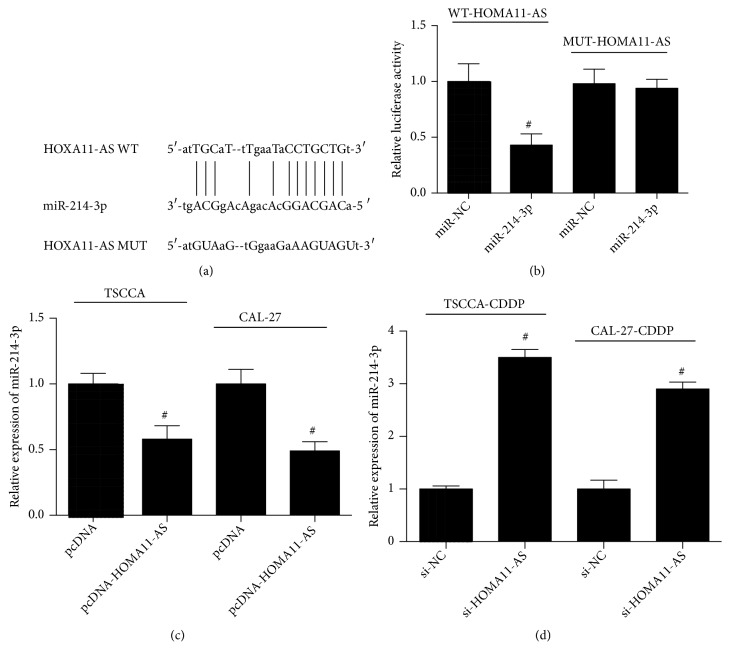
*HOMA11-AS interacted with and negatively regulated miR-214-3p in OSCC cells*. (a) Bioinformatics analysis indicated the putative binding sites and corresponding mutant region for HOMA11-AS within miR-214-3p. (b) Effect of miR-214-3p transfection on the luciferase activity of WT-HOMA11-AS and MUT-HOMA11-AS reporter systems was evaluated by dual luciferase reporter assay in 293T cells (p=0.037). (c) miR-214-3p expression in TSCCA-CDDP and CAL-27-CDDP cells transfected by si-HOMA11-AS or its negative control (p=0.039; p=0.031). (d) miR-214-3p expression in TSCCA and CAL-27 cells transfected by pcDNA-HOMA11-AS or its negative control (p=0.011; p=0.014). #P < 0.05.

To investigate the mechanism of HOMA11-AS-associated CDDP-resistance in OSCC cells, we explored the possible targets of HOMA11-AS regulation. Using bioinformatics analysis, we predicted a putative binding site of miR-214-3p in 3'-UTR of HOMA11-AS (Figure 3(a)). Then, we used luciferase reporter assay to evaluate the direct interaction between HOMA11-AS and miR-214-3p. We showed that transfection miR-214-3p mimics significantly decreased WT-HOMA11-AS luciferase activity, but not MUT-HOMA11-AS (Figure 3(b)), confirming the binding of predicted sites in HOMA11-AS by miR-214-3p. Moreover, upregulation of HOMA11-AS significantly inhibited the expression of miR-214-3p in CDDP-resistant TSCCA and CAL-27 cells (Figure 3(c)). Downregulation of HOMA11-AS in CDDP-resistant OSCC cells resulted in a significant increase of miR-214-3p expression (Figure 3(d)). These results demonstrated that HOMA11-AS interacted with and negatively regulated miR-214-3p in OSCC cells.

### 3.4. Inhibition of miR-214-3p Suppressed the Effect of HOMA11-AS Knockdown on Proliferation, CDDP Chemosensitivity, and Apoptosis in CDDP-Resistant OSCC Cells

**Figure 4:**
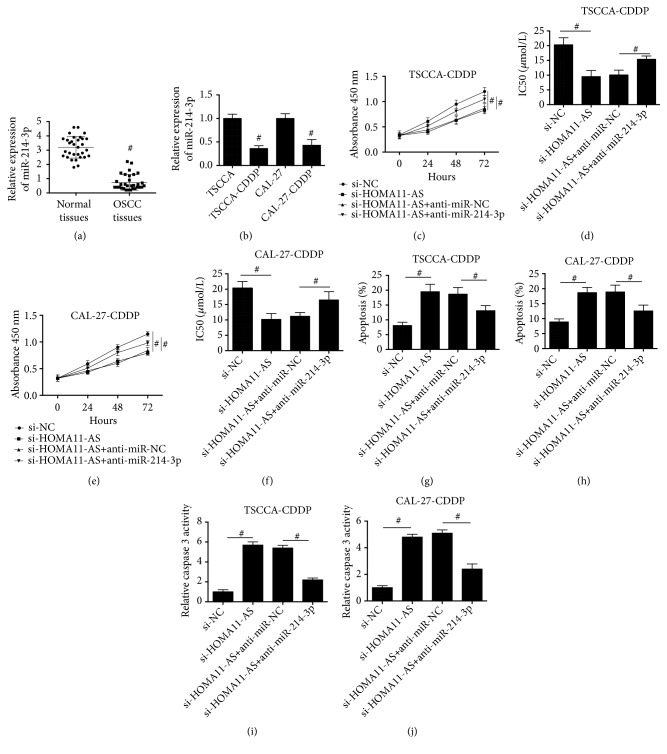
*Inhibition of miR-214-3p suppressed the effect of HOMA11-AS knockdown on proliferation, CDDP chemosensitivity, and apoptosis in CDDP-resistant OSCC cells*. (a) mRNA expression level of miR-214-3p in tumor tissues and corresponding noncancerous tissues of 31 OSCC patients (p=0.012). (b) mRNA expression level of miR-214-3p in OSCC cell lines (TSCCA and CAL-27) and their corresponding CDDP-resistant cell lines (TSCCA-CDDP and CAL-27-CDDP) (p=0.004; p=0.007). TSCCA-CDDP and CAL-27-CDDP cells were cotransfected with si-HOMA11-AS and anti-miR-214-3p or their negative controls. ((c)–(f)) Cell proliferation and IC50 value of CDDP were determined by CCK8 assay kit ((c) p=0.013; p=0.027; (d) p=0.009; p=0.022; (e) p=0.026; p=0.031; (f) p=0.013; p=0.038). ((g) and (h)) Apoptosis was evaluated by TUNEL assay ((g) p=0.028; p=0.032; (h) p=0.029; p=0.027). ((i) and (j)) Caspase 3 activities were detected using assay kit ((i) p=0.012; p=0.015; (j) p=0.019; p=0.016). #P < 0.05.

The expression pattern of miR-214-3p in OSCC tissues was examined and the results showed that miR-214-3p expression in OSCC clinical tissues was significantly lower than that in adjacent normal tissues (Figure 4(a)). Moreover, we compared the expression of miR-214-3p between the CDDP-sensitive and resistant OSCC cells.  Figure 4(b) showed that compared with CDDP-sensitive state, miR-214-3p expression was decreased when the cells were CDDP-resistant. To investigate whether miR-214-3p was the downstream target of HOMA11-AS-induced CDDP resistance in OSCC, we knocked down the expression of miR-214-3p in CDDP-resistant TSCCA and CAL-27 cells. The results showed that downregulation of miR-214-3p significantly suppressed si-HOMA11-AS-induced decrease of cell proliferation (Figures 4(c) and 4(e)) and reduction of IC50 value of CDDP (Figures 4(d) and 4(f)) in CDDP-resistant TSCCA and CAL-27 cells. Moreover, si-HOMA11-AS-induced increase of apoptosis (Figures 4(g) and 4(h)) and caspase 3 activities (Figures 4(i) and 4(j)) was notably inhibited by downregulation of miR-214-3p in CDDP-resistant TSCCA and CAL-27 cells. The results suggested that upregulation of miR-214-3p was involved in si-HOMA11-AS-induced decrease of proliferation and increase of apoptosis in CDDP-resistant OSCC cells.

### 3.5. PIM1 Was a Downstream Target of HOMA11-AS and miR-214-3p in the Regulation of CDDP Chemosensitivity in CDDP-Resistant OSCC Cells

**Figure 5:**
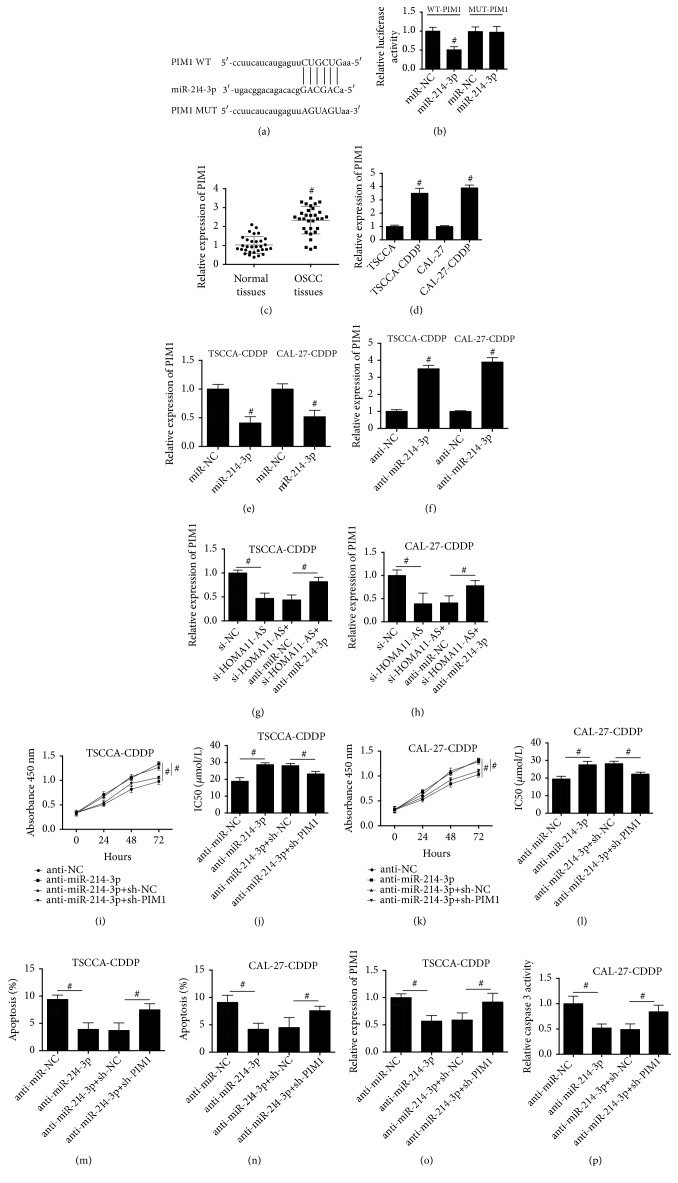
*PIM1 was a downstream target of HOMA11-AS and miR-214-3p in the regulation of CDDP chemosensitivity in CDDP-resistant OSCC cells*. (a) Bioinformatics analysis indicated the putative binding sites and corresponding mutant region for PIM1 within miR-214-3p. (b) Effect of miR-214-3p transfection on the luciferase activity of WT-PIM1 and MUT-PIM1 reporter systems was evaluated by dual luciferase reporter assay in 293T cells (p=0.029). (c) mRNA expression level of PIM1 in tumor tissues and corresponding noncancerous tissues of 31 OSCC patients (p=0.015). (d) mRNA expression level of PIM1 in OSCC cell lines (TSCCA and CAL-27) and their corresponding CDDP-resistant cell lines (TSCCA-CDDP and CAL-27-CDDP) (p=0.022; p=0.018). ((e) and (f)) TSCCA-CDDP and CAL-27-CDDP cells were transfected with miR-214-3p, or anti- miR-214-3p, or their negative controls. mRNA expression level of PIM1 was determined using real-time PCR ((e) p=0.028; p=0.032; (f) p=0.012; p=0.019). ((g) and (h)) TSCCA-CDDP and CAL-27-CDDP cells were cotransfected with si-HOMA11-AS and anti-miR-214-3p or their negative controls. mRNA expression level of PIM1 was determined using real-time PCR ((g) p=0.031; p=0.036; (h) p=0.037; p=0.041). ((i)–(p)) TSCCA-CDDP and CAL-27-CDDP cells were cotransfected with anti-miR-214-3p and sh-PIM1 or their negative controls. ((i)–(l)) Cell proliferation and IC50 value of CDDP were determined by CCK8 assay kit ((i) p=0.031; p=0.036; (j) p=0.031; p=0.039; (k) p=0.035; p=0.041; (l) p=0.022; p=0.023). ((m) and (n)) Apoptosis was evaluated by TUNEL assay ((m) p=0.021; p=0.027; (n) p=0.022; p=0.031). ((o) and (p)) Caspase 3 activities were detected using assay kit ((o) p=0.025; p=0.033; (p) p=0.026; p=0.032). #P < 0.05.

In the next step, we investigated the downstream target of miR-214-3p in the regulation of CDDP resistance in OSCC cells. Using bioinformatics analysis, we found that there was a putative binding site of miR-214-3p in the 3'-UTR of proto-oncogene serine/threonine-protein kinase (PIM-1) (Figure 5(a)). The luciferase reporter assay confirmed that miR-214-3p negatively regulated WT-PIM-1, but not MUT-PIM-1 (Figure 5(b)). The expression pattern of PIM-1 in OSCC tissues was examined and the results showed that PIM-1 expression in OSCC clinical tissues was significantly higher than that in adjacent normal tissues (Figure 5(c)). Moreover, we compared the expression of PIM-1 between the CDDP-sensitive and resistant OSCC cells.  Figure 5(d) showed that compared with CDDP-sensitive state, PIM-1 expression was increased when the cells were CDDP-resistant. Additionally, upregulation of miR-214-3p decreased PIM-1 expression (Figure 5(e)), while downregulation of miR-214-3p increased PIM-1 expression (Figure 5(f)) in CDDP-resistant TSCCA and CAL-27 cells. Downregulation of HOMA11-AS decreased PIM-1 expression, whose effect was inhibited by anti-miR-214-3p in CDDP-resistant TSCCA and CAL-27 cells (Figures 5(g) and 5(h)). Downregulation of PIM-1 significantly suppressed anti-miR-214-3p-induced increase of cell proliferation (Figures 5(i) and 5(k)) and increase of IC50 value of CDDP (Figures 5(j) and 5(l)) in CDDP-resistant TSCCA and CAL-27 cells. Moreover, anti-miR-214-3p-induced decrease of apoptosis (Figures 5(m) and 5(n)) and caspase 3 activities (Figures 5(o) and 5(p)) was notably inhibited by downregulation of PIM-1 in CDDP-resistant TSCCA and CAL-27 cells. The results suggested that PIM-1 was a target of HOMA11-AS/miR-214-3p signaling and upregulation of PIM-1 was involved in anti-miR-214-3p-induced increase of proliferation and decrease of apoptosis in CDDP-resistant OSCC cells.

### 3.6. Knockdown of HOMA11-AS Enhanced CDDP-Mediated Tumor Inhibition and Regulated miR-214-3p and PIM1 in Xenograft Mice

**Figure 6:**
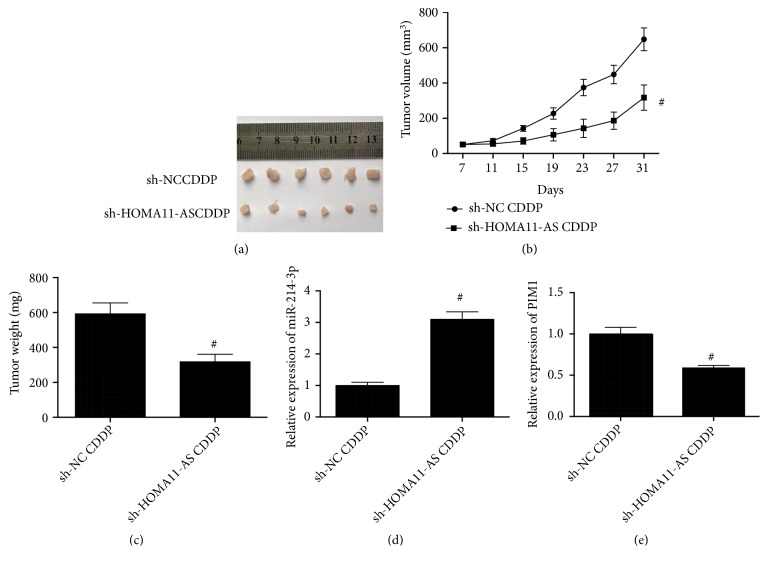
*Knockdown of HOMA11-AS enhanced CDDP-mediated tumor inhibition and regulated miR-214-3p and PIM1 in xenograft mice*. TSCCA-CDDP cells infected with sh-HOMA11-AS or its negative control were injected into nude mice to establish xenograft mice. (a) Images of tumor nodes. (b) Tumor volume was measured at indicated time points (p=0.008). (c) Tumor weight was analyzed (p=0.007). (d) miR-214-3p expression in tumor tissues (p=0.021). (e) PIM1 expression in tumor tissues (p=0.034). #P < 0.05.

We then tested the role of HOMA11-AS in CDDP resistance in xenograft mice in vivo. Knockdown of HOMA11-AS significantly decreased the growth of tumor weight and volume in response to CDDP injection in xenograft mice (Figures 6(a), 6(b), and 6(c)). Moreover, in tumor nodes, knockdown of HOMA11-AS markedly increased the expression of miR-214-3p and reduced the expression of PIM1. The results confirmed the promotive role of HOMA11-AS in CDDP resistance and provided in vivo evidence for the regulation of miR-214-3p and PIM1 by HOMA11-AS in OSCC tumors.

## 4. Discussion

Chemoresistance tremendously hinder therapeutic effect of chemotherapy for tumor and limits the prognosis of tumor patients [7]. Accumulating evidence supports that dysregulation of lncRNAs participates in primary or acquired chemoresistance through various molecular mechanisms [24]. It is recently recognized that lncRNAs plays an important role in drug resistance in OSCC. HOXA11-AS has been found to function as an oncogenic or tumor promotive regulator in several types of human cancers [15–21]. Moreover, HOXA11-AS was identified to drive CDDP resistance of human lung cancer [23]. We proposed that HOXA11-AS dysregulation was involved in CDDP resistance in OSCC.

To test the hypothesis, we compared the expression pattern of HOXA11-AS between CDDP-sensitive and resistant condition and evaluated the role of HOXA11-AS using a battery of in vitro and in vivo models. HOXA11-AS expression was increased in OSCC clinical tissues and cells and upregulated under CDDP-resistant condition. Upregulation of HOXA11-AS promoted proliferation in CDDP-sensitive OSCC cells and inhibited CDDP-induced cytotoxicity, as evidenced by increased IC50 value. In contrast, downregulation of HOXA11-AS decreased proliferation in CDDP-resistant OSCC cells and increased CDDP-induced cytotoxicity, as illustrated by increased IC50 value. Moreover, the required role of HOXA11-AS in CDDP resistance was testified in xenograft tumor mice in vivo. Downregulation of HOXA11-AS increased the percentage of apoptotic cell and caspase 3 activities in CDDP-resistant OSCC cells, indicating that inhibition of apoptosis may be involved in HOXA11-AS-induced facilitation of CDDP-resistance in OSCC. Based on these findings, we provide further evidence for the positive role of HOXA11-AS in tumor development and, for the first time, reported the key role of HOXA11-AS in CDDP-resistance in OSCC.

LncRNAs/miRNAs interaction is an important mechanism for the biological function of HOXA11-AS in tumor regulation [25–27]. To explore the mechanism of HOXA11-AS-induced regulation of CDDP-resistance in OSCC, we used bioinformatics analysis and found a candidate miRNA, miR-214-3p. miR-214-3p expression was decreased in OSCC clinical tissues and was reduced in CDDP-resistant cells. Further experiments, using luciferase reporter assay and plasmid transfection, confirmed the negative regulation of these two molecules by each other. Furthermore, downregulation of miR-214-3p inhibited si-HOXA11-AS-induced regulation of proliferation and apoptosis in CDDP-resistant OSCC cells. Dysregulation of miR-214-3p has been previously reported to be involved in tumor development and miR-214-3p is mainly found to play a tumor-suppressive role. For instance, overexpression of miR-214-3p in esophageal squamous cancer cells enhanced sensitivity to CDDP by targeting survivin directly and indirectly through CUG-BP1 [28]. miR-214-3p has been found to improve the overall survival prediction of muscle-invasive bladder cancer patients after radical cystectomy [29]. In particular, in glioma, HOXA11-AS functioned as a competing endogenous RNA (ceRNA) for miR-214-3p, which in turn positively regulated the expression of its direct target EZH2 [30]. According to these findings and our results, we proposed that HOXA11-AS/miR-214-3p may be a ubiquitous pathway in the regulation of tumor development and malignancy.

Considering the pivotal role of lncRNAs acting as the ceRNAs to repress target mRNAs expression by sequestering miRNAs, we tried out to find the downstream target of miR-214-3p that may be responsible for the biological role of HOXA11-AS in CDDP-resistance in OSCC. Bioinformatics analysis indicated that miR-214-3p could bind with the 3'UTR of PIM1 whose effect was confirmed by luciferase reporter assay. Loss and gain of function assay indicated that miR-214-3p could negatively and HOXA11-AS could positively regulate PIM1. Downregulation of PIM1 could suppress anti-miR-214-3p-induced regulation of proliferation and apoptosis in CDDP-resistant OSCC cells, suggesting that PIM1 was the downstream target of HOXA11-AS/miR-214-3p pathway.

In conclusion, we identified that HOXA11-AS facilitated CDDP-resistance in OSCC and clarified that miR-214-3p/PIM1 was the downstream target of HOXA11-AS function. The findings highlight the importance of HOXA11-AS/miR-214-3p/PIM1 axis in the drug resistance of OSCC and provide potential targets for improving chemotherapy of OSCC.

## Data Availability

The data used to support the findings of this study are available from the corresponding author upon request.

## References

[1] Montero P. H., Patel P. D., Palmer F. L. (2012). Changing trends in smoking and alcohol consumption in patients with oral cancer treated at Memorial Sloan-Kettering Cancer Center from 1985 to 2009. *Archives of Otolaryngology—Head and Neck Surgery*.

[2] Rao S. V. K., Mejia G., Roberts-Thomson K., Logan R. (2013). Epidemiology of oral cancer in Asia in the past decade - An update (2000-2012). *Asian Pacific Journal of Cancer Prevention*.

[3] Warnakulasuriya S. (2009). Global epidemiology of oral and oropharyngeal cancer. *Oral Oncology*.

[4] Ng J. H., Iyer N. G., Tan M.-H., Edgren G. (2017). Changing epidemiology of oral squamous cell carcinoma of the tongue: A global study. *Head & Neck*.

[5] Sacco A. G., Cohen E. E. (2015). Current treatment options for recurrent or metastatic head and neck squamous cell carcinoma. *Journal of Clinical Oncology*.

[6] Kessler P., Grabenbauer G., Leher A., Bloch-Birkholz A., Vairaktaris E., Neukam F. W. (2008). Neoadjuvant and adjuvant therapy in patients with oral squamous cell carcinoma. Long-term survival in a prospective, non-randomized study. *British Journal of Oral and Maxillofacial Surgery*.

[7] Vermorken J. B., Mesia R., Rivera F. (2008). Platinum-based chemotherapy plus cetuximab in head and neck cancer. *The New England Journal of Medicine*.

[8] Wang K. C., Chang H. Y. (2011). Molecular mechanisms of long noncoding RNAs. *Molecular Cell*.

[9] Yang L., Froberg J. E., Lee J. T. (2014). Long noncoding RNAs: Fresh perspectives into the RNA world. *Trends in Biochemical Sciences*.

[10] Huarte M. (2015). The emerging role of lncRNAs in cancer. *Nature Medicine*.

[11] Schmitt A. M., Chang H. Y. (2016). Long noncoding RNAs in cancer pathways. *Cancer Cell*.

[12] Khaitan D., Dinger M. E., Mazar J. (2011). The melanoma-upregulated long noncoding RNA *SPRY4-IT1* modulates apoptosis and invasion. *Cancer Research*.

[13] Fatica A., Bozzoni I. (2014). Long non-coding RNAs: new players in cell differentiation and development. *Nature Reviews Genetics*.

[14] Yang X., Song J. H., Cheng Y. (2014). Long non-coding RNA HNF1A-AS1 regulates proliferation and migration in oesophageal adenocarcinoma cells. *Gut*.

[15] Xue J. Y., Huang C., Wang W., Li H., Sun M., Xie M. (2018). HOXA11-AS: a novel regulator in human cancer proliferation and metastasis. *OncoTargets and Therapy*.

[16] Mu S., Ai L., Fan F., Sun C., Hu Y. (2018). Prognostic and clinicopathological significance of long noncoding RNA HOXA11-AS expression in human solid tumors: A meta-analysis. *Cancer Cell International*.

[17] Mills E. L., Pierce K. A., Jedrychowski M. P. (2018). Accumulation of succinate controls activation of adipose tissue thermogenesis. *Nature*.

[18] Lu C.-W., Zhou D.-D., Xie T. (2018). HOXA11 antisense long noncoding RNA (HOXA11-AS): A promising lncRNA in human cancers. *Cancer Medicine*.

[19] Yu W., Peng W., Jiang H., Sha H., Li J. (2017). LncRNA HOXA11-AS promotes proliferation and invasion by targeting miR-124 in human non–small cell lung cancer cells. *Tumor Biology*.

[20] Yu J., Hong J.-F., Kang J., Liao L.-H., Li C.-D. (2017). Promotion of LncRNA HOXA11-AS on the proliferation of hepatocellular carcinoma by regulating the expression of LATS1. *European Review for Medical and Pharmacological Sciences*.

[21] Lu Q., Zhao N., Zha G., Wang H., Tong Q., Xin S. (2017). LncRNA HOXA11-AS exerts oncogenic functions by repressing p21 and miR-124 in uveal melanoma. *DNA and Cell Biology*.

[22] Qu L., Jin M., Yang L. (2018). Expression of long non-coding RNA HOXA11-AS-as is correlated with progression of laryngeal squamous cell carcinoma. *American Journal of Translational Research*.

[23] Zhao X., Li X., Zhou L. (2018). LncRNA HOXA11-AS drives cisplatin resistance of human LUAD cells via modulating miR-454-3p/Stat3. *Cancer Science*.

[24] Malhotra A., Jain M., Prakash H., Vasquez K. M., Jain A. (2017). The regulatory roles of long non-coding RNAs in the development of chemoresistance in breast cancer. *Oncotarget *.

[25] Lin Z., Sun L., Xie S. (2018). Chemotherapy-Induced Long Non-coding RNA 1 Promotes Metastasis and Chemo-Resistance of TSCC via the Wnt/*β*-Catenin Signaling Pathway. *Molecular Therapy*.

[26] Tan D. S. W., Chong F. T., Leong H. S. (2017). Long noncoding RNA EGFR-AS1 mediates epidermal growth factor receptor addiction and modulates treatment response in squamous cell carcinoma. *Nature Medicine*.

[27] Ding L., Sousa K. M., Jin L. (2016). Vertical sleeve gastrectomy activates GPBAR-1/TGR5 to sustain weight loss, improve fatty liver, and remit insulin resistance in mice. *Hepatology*.

[28] Phatak P., Byrnes K. A., Mansour D. (2016). Overexpression of miR-214-3p in esophageal squamous cancer cells enhances sensitivity to cisplatin by targeting survivin directly and indirectly through CUG-BP1. *Oncogene*.

[29] Ecke T. H., Stier K., Weickmann S. (2017). miR-199a-3p and miR-214-3p improve the overall survival prediction of muscle-invasive bladder cancer patients after radical cystectomy. *Cancer Medicine*.

[30] Xu C., He T., Li Z., Liu H., Ding B. (2017). Regulation of HOXA11-AS/miR-214-3p/EZH2 axis on the growth, migration and invasion of glioma cells. *Biomedicine & Pharmacotherapy*.

